# SUMOylation in Control of Accurate Chromosome Segregation during Mitosis

**DOI:** 10.2174/138920312802430563

**Published:** 2012-08

**Authors:** Jun Wan, Divya Subramonian, Xiang-Dong Zhang

**Affiliations:** Department of Biological Sciences, Wayne State University, Detroit, Michigan 48202, USA

**Keywords:** Centromere, chromosome segregation, fibrous corona, kinetochore, mitosis, SUMOylation, SUMO interacting motif (SIM), spindle assembly checkpoint (SAC).

## Abstract

Posttranslational protein modification by small ubiquitin-related modifier (SUMO) has emerged as an important regulatory mechanism for chromosome segregation during mitosis. This review focuses on how SUMOylation regulates the centromere and kinetochore activities to achieve accurate chromosome segregation during mitosis. Kinetochores are assembled on the specialized chromatin domains called centromeres and serve as the sites for attaching spindle microtubule to segregate sister chromatids to daughter cells. Many proteins associated with mitotic centromeres and kinetochores have been recently found to be modified by SUMO. Although we are still at the early stage of elucidating how SUMOylation controls chromosome segregation during mitosis, a substantial progress has been achieved over the past decade. Furthermore, a major theme that has emerged from the recent studies of SUMOylation in mitosis is that both SUMO conjugation and deconjugation are critical for kinetochore assembly and disassembly. Lastly, we propose a model that SUMOylation coordinates multiple centromere and kinetochore activities to ensure accurate chromosome segregation.

## INTRODUCTION

Accurate chromosome segregation during mitosis is essential to ensure that each daughter cell receives a copy of every chromosome and to prevent aneuploidy, a major driver of tumorigenesis [1-3]. To achieve the accurate chromosome segregation during mitosis, large protein complexes known as kinetochores need to be properly assembled on the centromeric regions of duplicated chromosomes. The centromere is a chromatin region containing a histone H3 variant called centromeric protein A (CENP-A) and functions as the docking site for kinetochore assembly [4]. The kinetochore is one of the most complex cellular substructures [5] and contains over 130 different proteins in vertebrates [6]. Kinetochores coordinate three major activities during mitosis: 1) they attach two sister-chromatids to the plus ends of microtubules from the opposite spindle poles, 2) they use the spindle assembly checkpoint (SAC) to prevent the onset of anaphase until all sister-chromatids are bioriented at the metaphase plate, and 3) they generate force for chromosome movements [7].

Accumulating evidence has shown that the SUMO modification pathway plays a pivotal role in regulation of chromosome segregation [8-10]. Early genetic studies in yeast indicate that both SUMO conjugation and deconjugation are essential for chromosome condensation, sister chromatid cohesion, centromere and kinetochore function, and chromosome segregation [11-17]. For instance, overexpression of either SUMO (Smt3) or a SUMO-specific protease (Ulp2/Smt4) in *Saccharomyces cerevisiae* suppresses the temperature sensitive phenotype of the mutant allele of *MIF2*, which encodes a homolog of the mammalian centromere protein CENP-C [11]. This finding provides the first genetic evidence that SUMOylation and deSUMOylation are involved in the centromere and kinetochore function. Since then, numerous centromere and kinetochore proteins have been identified as SUMO targets. Here we will first introduce the SUMO pathway and then focus on reviewing the roles of SUMOylation in control of the activities and functions of centromeres and kinetochores to ensure accurate chromosome segregation during mitosis.

## THE SUMO PATHWAY

The small ubiquitin-related modifiers (SUMOs) are reversibly conjugated to hundreds of different proteins and therefore regulate many cellular processes including cell cycle, nucleocytoplasmic transport, DNA repair, gene expression, and protein stability [8,18-23]. Consistent with the essential roles of the SUMO pathway in many biological processes, dysregulation of SUMOylation has been implicated to human diseases including various types of cancer and multiple neurodegenerative diseases [24]. Since the mammalian Ran GTPase activating protein RanGAP1 was identified as the first SUMO substrate over 15 years ago [25,26], many exciting advances in our understanding of the SUMO pathway have been accomplished in this rapid developing field [8,19,22,27,28]. Currently, the fundamental molecular mechanisms for SUMO conjugation and deconjugation have been relatively well established. At the same time, not only many SUMO substrates have been identified but also the effects of SUMOylation on these substrates have been elucidated in multiple model systems and organisms, including budding yeast, fission yeast, mammalian culture cells, *Xenopus* and *Arabidopsis*. 

**Fig. (1):**
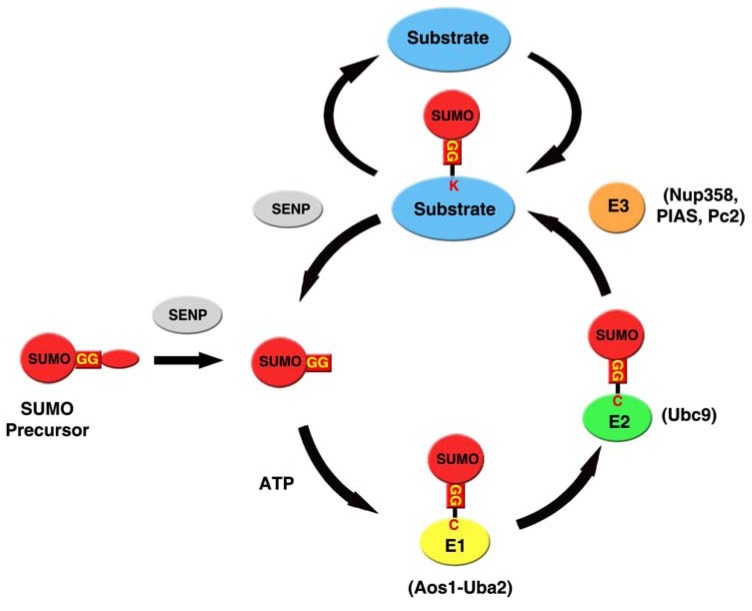
The SUMO pathway. The SUMO precursor is processed by SUMO-specific isopeptidases called SENPs in vertebrates to expose its C-terminal double-glycine (GG) motif. The mature SUMO is activated by the E1 activating enzyme (Aos1-Uba2) to form a thioester bond between the C-terminal glycine residue of SUMO and the cysteine (C) residue of the E1 subunit Uba2. SUMO is then transferred to the catalytic cysteine (C) residue of the E2 conjugating enzyme called Ubc9. Finally, SUMO is transferred from Ubc9 to the substrate by forming an isopeptide bond between the C-terminal glycine residue (G) of SUMO and a lysine (K) residue of the substrate. The last step is usually facilitated by a SUMO E3 ligase that promotes SUMO conjugation to specific protein substrates. SUMO is deconjugated for its substrates by SENP isopeptidases (SENPs).

Although a single SUMO protein is expressed in yeast and invertebrates, vertebrates express three SUMO proteins: SUMO-1, SUMO-2 and SUMO-3 [18]. In human, SUMO-2 and SUMO-3 share ~96% identity with each other and are thus referred to collectively as SUMO-2/3, whereas the identity between SUMO-2/3 and SUMO-1 is less than 45%. All SUMOs are expressed as the SUMO precursor proteins that must be posttranslationally processed to expose their C-terminal double-glycine (GG) motifs to generate the mature SUMOs for their conjugation to protein targets (Fig. **1**) [8,18,19]. This processing step is catalyzed by the SUMO isopeptidases, called ubiquitin like protein proteases (Ulps) in yeast [14] and Sentrin-specific proteases (SENPs) in vertebrates (Fig. **1**) [29]. Like ubiquitination, SUMOylation requires a similar enzymatic cascade that involves three classes of enzymes. In an ATP-dependent reaction, the mature SUMO is first activated by the SUMO E1 activating enzyme (Aos1-Uba2) and a thioester bond is formed between the C-terminal glycine residue of SUMO and the cysteine (C) residue of the E1 enzyme (Fig. **1**) [30]. SUMO is then transferred to the catalytic cysteine (C) residue of the SUMO E2 conjugating enzyme (Ubc9) (Fig. **1**) [31-34]. Finally, SUMO is transferred from Ubc9 to the substrate by forming an isopeptide bond between the C-terminal glycine residue (G) of SUMO and a lysine (K) residue of the substrate (Fig. **1**). The last step is usually facilitated by an E3 ligase that promotes SUMO conjugation to specific proteins (Fig. **1**) [8,18,35,36]. 

Different from the ubiquitin E2 enzymes, Ubc9 plays a direct role in recognition of protein substrates through its interaction with the SUMOylation consensus sequence Ψ-K-x-[E/D] in the substrates, where Ψ is a hydrophobic amino acid residue, K is the lysine residue for SUMO conjugation, x is any amino acid, and E/D represents either a glutamic acid (E) or an aspartic acid (D) [37-39]. Interestingly, recent studies have shown that some SUMOylation sites contain an inverted SUMOylation consensus sequence [E/D]-x-K-Ψ [40]. In many cases, the SUMOylation consensus sequence Ψ-K-x-[E/D] also contains an adjacent motif to enhance its SUMO conjugation. The extended SUMOylation consensus sequence motifs include the negatively charged amino acid-dependent SUMOylation motif (NDSM) with extra negatively charged amino acids in close proximity to its core SUMOylation consensus sequence, the hydrophobic cluster SUMOylation motif (HCSM) with a cluster of hydrophobic amino acids, and the phosphorylation-dependent SUMOylation motif (PDSM) with an adjacent serine (S) residue for phosphorylation [40,41]. On the other hand, many SUMO conjugations occur at the non-consensus sequences, whereas many non-SUMOylated proteins also contain the SUMOylation consensus sequences. Therefore, SUMOylation sites must be directly determined and confirmed by *in vitro* and/or *in vivo* experiments.

Although SUMO E1 enzyme and Ubc9 alone are sufficient for *in vitro* SUMO modification of many known substrates, SUMO E3 ligases play an important regulatory role *in vivo* by increasing the SUMOylation efficiency and also by determining the substrate specificity. Based upon their evolutionary conservation, the current known SUMO E3 ligases can be classified into two main groups. A conservative group of E3 ligases has been found in all eukaryotes and contains a RING-finger like domain called SP-RING domain, which is responsible for recruiting Ubc9 [8,18,19,36,42]. The SP-RING E3 ligases include the PIAS (protein inhibitor of activated STAT) family proteins (PIAS1, PIAS3, PIASxα, PIASxβ and PIASy) in vertebrates and the Siz family proteins (Siz1 and Siz2) in *Saccharomyces cerevisiae *[8,18,19,36]. The Siz1 and Siz2 are required for most SUMO conjugation in budding yeast [43,44]. In yeast and human, the SUMO E3 ligase Mms21 also contains a SP-RING domain and plays an essential role in DNA repair [45,46]. Furthermore, the yeast SP-RING E3 ligase Zip3 regulates the assembly of synaptonemal complex during meiosis [47]. On the other hand, the human PIAS-like E3 ligase hZimp10 is capable to stimulate the SUMOylation of androgen receptor (AR), leading to an increase of transcription activity of AR [48]. In contrast to the conservative group of SP-RING E3 ligases, the non-conservative group of E3 ligases is vertebrate-specific and has no obvious yeast homologues. These vertebrate-specific E3 ligases include the nucleoporin Nup358/RanBP2 [49], the polycomb-group protein Pc2 [50], the histone deacetylases including HDAC4 [51,52] and HDAC7 [53], and the topoisomerase I-binding protein Topors [54-56].

DeSUMOylation is essential to ensure the reversible nature of SUMO conjugation [8,18,19,27,57]. SUMO isopeptidases (Ulps/SENPs) are responsible for both processing the SUMO precursors and deconjugating the SUMOs from their protein targets (Fig. **1**) [14,15,27]. Budding yeast has two SUMO isopeptidases (Ulp1 and Ulp2/Smt4) [27,57]. While Ulp1 is associated with the nuclear pore complex (NPC) [14,15,58], Ulp2 has a distribution throughout the nucleus [15]. Ulp1 is responsible for processing the SUMO precursors and also essential for the cell cycle progression through the G2/M phase [14,59,60]. Although Ulp2 is not essential for vegetative growth, it is crucial for meiosis [15]. During mitosis, the Ulp2 is preferentially required for sister-chromatid cohesion at centromere regions, and the defects in Ulp2 leads to the precocious loss of centromeric cohesion [61]. Interestingly, Ulp2 is also specifically required for disassembly of polymeric SUMO chains [15,62]. The distinct subcellular localizations of Ulp1 and Ulp2 are important to determine their substrate specificities [63]. On the other hand, there are six different isopeptidases (SENP1, SENP2, SENP3, SENP5, SENP6, and SENP7) in human cells [27,57]. The six SENPs share a common C-terminal catalytic domain but have distinct N-terminal domains, which are critical for their subcellular localizations and substrate specificities [27,57]. The sequence alignment of the human SENPs and the budding yeast Ulps suggests that SENP1, 2, 3 and 5 belong to the Ulp1-like branch, whereas SENP6 and 7 fall into the Ulp2-like branch [27]. SENP1 and SENP2 are most closely related to each other and catalyze both processing and deconjugation of SUMO-1 and SUMO-2/3 [64-66]. In addition, both SENP1 and SENP2 are associated with the nuclear pore complex (NPC) and have a distribution throughout the nucleus [67-70]. Among the six SENPs, SENP3 and SENP5 are most closely related with each other and localize to the nucleolus with a preference for SUMO-2/3 processing and deconjugation [71-73]. Similarly to Ulp2, SENP6 and SENP7 localize throughout the nucleoplasm and have a strong preference for disassembly of SUMO-2/3 polymeric chains [74,75]. The different sub-cellular localization of SUMO isopeptidases may determine their accessibilities to discrete SUMO targets and therefore their substrate specificities. 

## THE SUMO SIGNALS AT CENTROMERES AND KINETOCHORES

**Fig. (2):**
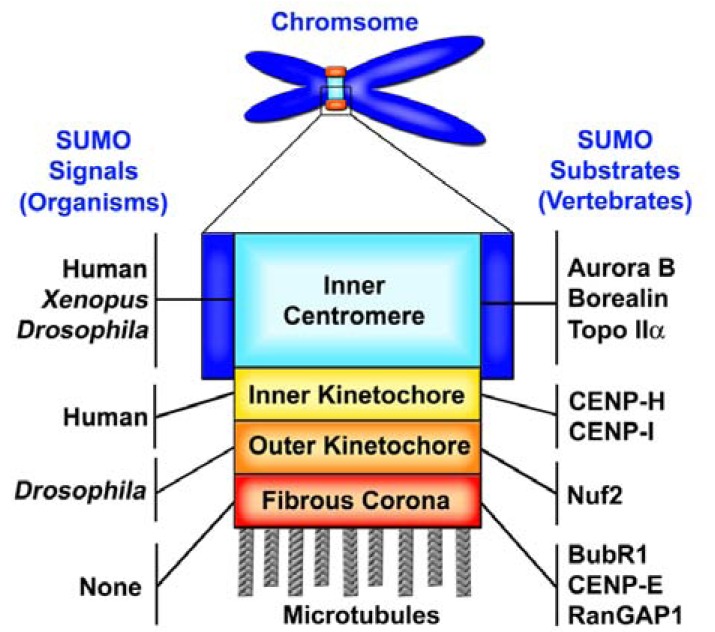
Both SUMO signals and known SUMO substrates are associated with the mitotic centromere and kinetochore of a condensed chromosome. The enlarged region of the mitotic chromosome represents inner centromere, inner kinetochore, outer kinetochore, and fibrous corona. The exact localizations of SUMO signals at centromeres and kinetochores in human HeLa cells, *Xenopus* egg extracts and *Drosophila* culture cells are determined by fluorescence or immunofluorescence microscopy (on the left). The known vertebrate SUMO targets, which have been identified and confirmed *in vivo*, are schematically represented here (on the right) and also described in Table **1**

The subcellular localization of SUMO at mitotic centromeres and kinetochores has been extensively analyzed by fluorescence microscopy in different organisms including human, *Xenopus* and* Drosophila *[76-78] (Fig. **2**). The mitotic centromere and kinetochore can be structurally divided into four distinct regions including inner centromere, inner kinetochore, outer kinetochore and fibrous corona [8,79,80] (Fig. **2**). Here we briefly summarize the key findings about the SUMO signals at centromeres and kinetochores during mitosis [76-78] (Fig. **2**). 

**1)** Immunofluorescence microscopy analysis using antibodies specific to SUMO-2/3 showed that SUMO-2/3 signals are concentrated to many distinct foci on the condensed chromosomes from prophase to metaphase and eventually coat the entire chromosomes during anaphase and telophase in mammalian cells [76]. During metaphase, the SUMO-2/3 foci on the chromosomes are significantly co-localized with the inner centromere protein CENP-B and partially overlapped with the inner kinetochore protein CENP-C [76] (Fig. **2**). In contrast, the SUMO-1 signals are localized to the mitotic spindle during metaphase and later concentrated to the spindle midzone during anaphase and telophase [25,76,81]. These results support a model that SUMO-1 and SUMO-2/3 paralogs are conjugated to different subsets of proteins at distinct subcellular localizations and therefore regulate discrete mitotic processes in vertebrates [76]. **2) **In *Xenopus* egg extracts, the EGFP-SUMO-2 signals are co-localized with Aurora B at inner centromeres of condensed chromosomes [77] (Fig. **2**). This may simply reflect the fact that the topoisomerase IIα (Topo IIα), which is concentrated at the inner centromere region, is the major SUMO-2/3 substrate in the mitotic *Xenopus* egg extracts [77,82]. **3)** In *Drosophila* cultured cells, SUMO conjugates are mainly localized to inner centromeres and outer kinetochore plates during prometaphase and are also targeted to the spindle midzone during anaphase. This result suggests that the single SUMO in invertebrates, such as *Drosophila*, plays the roles of both vertebrate SUMO-1 and SUMO-2/3 during mitosis [78] (Fig. **2**). 

In all the above organisms ranging from invertebrates to mammals, the SUMO signals have been observed at centromeres and kinetochores during the early stages of mitosis including prophase, prometaphase and metaphase [76-78] (Fig. **2**). Consistent with the finding that overexpression of the SUMO isopeptidase SENP2 leads to a loss of the SUMO-2/3 signals at centromeres and kinetochores, recent studies have demonstrated that many centromere and kinetochore proteins are *bona fide* SUMO substrates [76-78]. Therefore, we would like to consider that the SUMO signals detected at mitotic centromeres and kinetochores are mainly derived from the SUMO-modified proteins other than the free forms of SUMOs.

Three types of posttranslational modifications, including phosphorylation, ubiquitination and SUMOylation, have been demonstrated to play the essential roles in chromosome segregation during mitosis [8-10]. Interestingly, only “SUMOylation” signals have been reported to be directly detected at mitotic centromeres and kinetochores in both invertebrate and vertebrate cells [76-78] (Fig. **2**). These evolutionally conserved “SUMOylation” signals at mitotic centromeres and kinetochores are consistent with a model that SUMOylation functions as a master regulator of centromere and kinetochore activities during mitosis. Although the “SUMOylation” signals have not been directly detected in yeast, many centromere and kinetochore proteins have been identified as SUMO substrates in yeast, supporting a conserved role of SUMOylation in regulation of mitosis in all eukaryotes [8]. Consistent with the conserved role of SUMOylation in regulation of the centromere/kinetochore activities, SUMOs have been identified as suppressors of the temperature-sensitive mutants of the centromeric protein CENP-C in both yeast and chicken cells by genetic screenings [11,83]. 

## ROLES OF SUMO MODIFICATION AT CENTROMERES AND KINETOCHORES

Consistent with the observed SUMO signals at centromeres and kinetochores in both invertebrates and vertebrates (Fig. **2**), many centromere and kinetochore proteins have been identified as SUMO targets in yeast and vertebrates [8]. Since the yeast SUMO targets at centromeres and kinetochores have been extensively reviewed [8], we thereby mainly focus on reviewing the vertebrate SUMO targets associated with centromeres and kinetochores and also the roles of their SUMOylation in control of chromosome segregation. The precise localizations of these vertebrate SUMO targets at the centromere and kinetochore region are elucidated in Fig. **2** and Table **1**. Furthermore, the other information and properties of these SUMO targets, including their protein GI numbers, SUMOylation sites, SUMO-1 or SUMO-2/3-preferential modification, SUMOylation time during the cell cycle, associated protein complexes, and corresponding reference(s), are summarized in Table **1**.

### Topoisomerase IIα at Inner Centromere

Topoisomerase IIα (Topo IIα) has been identified as one of the first mitotic SUMO targets in budding yeast and vertebrates (Table **1** and Fig. **2**) [61,82]. During mitosis, Topo IIα is re-localized from chromosome arms to the centromeres of sister-chromatids [93,94]. One of the main functions of Topo IIα at the centromere is to decatenate the last major site of attachment between sister chromatids for chromosome segregation [95]. To ensure accurate chromosome segregation, each pair of sister-chromatids must be bioriented by the kinetochore-microtubules (kMTs) emanating from the opposite spindle poles and aligned at the metaphase plate, a plane equally distant from the two spindle poles. This correct bi-orientation of sister-chromatids leads to the silencing of the spindle assembly checkpoint (SAC), a sensing device that is active in prometaphase to prevent the precocious chromosome separation [96]. The silencing of the SAC activates the anaphase-promoting complex/cyclosome (APC/C), an E3 ubiquitin ligase, and leads to the APC/C-dependent polyubiquitination and proteasome-mediated degradation of securin. The degradation of securin then releases its inhibitory effect on separase, resulting in the separase-mediated cleavage of the cohesin complex that holds the sister-chromatid together. During the metaphase to anaphase transition, not only the cohesin-mediated cohesion but also the DNA catenation must be resolved to separate the sister-chromatids [97]. Accumulating evidence has indicated that SUMOylation plays a critical role in regulation of Topo IIα-mediated decatenation of centromeric DNA during mitosis [87,98]. 

In the budding yeast, the SUMOylated form of Topo II is accumulated in the Ulp2/Smt4-deletion strain (*ulp2Δ*), indicating that Ulp2/Smt4 is likely the SUMO-specific protease responsible for deSUMOylation of Topo II [61]. The yeast *ulp2Δ* mutants exhibit a prolonged metaphase and have defects in centromeric cohesion [61]. Furthermore, the cohesion defects in the yeast *ulp2Δ* strains can be significantly suppressed by the expression of a Topo II mutant lacking SUMOylation sites, suggesting that Topo II SUMOylation plays an important role in regulation of centromeric cohesion [61]. Currently, the SUMO-specific protease (SENP) responsible for deSUMOylation of Topo IIα in vertebrates has not been identified. Studies of SUMOylation in *Xenopus* egg extracts demonstrated that Topo IIα is one of the major SUMO-2/3 conjugates associated with mitotic chromosomes [82]. In *Xenopus *egg extracts, SUMO-2/3 modification of Topo IIα is at the highest level during metaphase and then rapidly disappears during anaphase, suggesting that this modification is tightly regulated during mitosis [82]. Global inhibition of SUMO conjugation by adding dominant-negative form of Ubc9 mutant proteins in* Xenopus* egg extracts leads to the failure of sister-chromatid segregation at the onset of anaphase, suggesting that SUMO-2/3 conjugation of Topo IIα and/or other substrates is required to the metaphase-anaphase transition [82].

PIASy has been found to be required for SUMO-2/3 modification of Topo IIα and also the accumulation of SUMO-2/3 conjugates at the inner centromere region of mitotic chromosomes in *Xenopus* extracts (Fig. **2**) [77]. In budding yeast, SUMOylation of Topo II is required for its targeting to pericentromeric regions and also controlled by Siz1 and Siz2, the yeast homologs of vertebrate PIASy [99]. In human cells, two studies have found that PIASy is required for SUMO-2/3 modification of Topo IIα and its localization to centromeres [100,101]. However, Nup358/RanBP2 has been found to be the SUMO E3 ligase for Topo IIα in mice [102]. In mouse embryonic fibroblast (MEF) cells with the reduced expression of Nup358/RanBP2, Topo IIα has a defect for its SUMOylation and fails to localize to inner centromeres during mitosis [102]. Recent studies by Ryu and Azuma have shown that the N-terminus of PIASy is responsible for the kinetochore localization of PIASy by interacting with Rod and Zw10, components of the RZZ complex at the kinetochore fibrous corona [96,103]. Furthermore, the kinetochore localization of PIASy is essential for the centromeric SUMO-2/3 modification of Topo IIα and other chromosome-associated proteins during mitosis [103]. Studies in *D. melanogaster *embryos, human cells, and *Xenopus *extracts have demonstrated that the Rod/Zw10/Zwilch (RZZ) complex at kinetochores plays an essential role in the SAC by recruiting two spindle checkpoint proteins Mad1/Mad2 to unattached kinetochores. Interestingly, PIASy-dependent SUMO-2/3 modification of Topo IIα significantly inhibits the decatenation activity of Topo IIα in *Xenopus* egg extracts [87]. Furthermore, Topo IIα is modified by SUMO-2/3 at the lysine 660 (K660) within the DNA gate domain involved in the DNA cleavage and re-ligation. The SUMO-2/3 modification of Topo IIα at the K660 is responsible for SUMOylation-mediated inhibition of Topo IIα, supporting a model that PIASy-dependent SUMOylation of Topo IIα functions in temporally preventing the resolution of centromeric DNA until the onset of anaphase [87].

### The CPC Complex at Inner Centromere

The chromosomal passenger complex (CPC) consists of the Aurora B kinase and three non-enzymatic subunits INCENP, Survivin and Borealin [104]. The CPC has a dynamic localization during mitosis. At the entry of mitosis, the CPC is initially localized to both chromosome arms and inner centromeres (Fig. **2**). As the cell cycle progression to prometaphase and metaphase, the CPC is mainly concentrated to the inner centromeres. Upon the sister-chromatid separation at the onset of anaphase, the CPC is re-localized from the inner centromeres to the spindle midzone [104]. The CPC plays a central role in correcting erroneous kinetochore-microtubule attachments during prometaphase, SAC, and cytokinesis [104,105]. Aurora B-dependent phosphorylation of some key kinetochore proteins including Ndc80/Hec1 has been demonstrated to reduce their binding affinity to microtubules, leading to the destabilization and also the correction of the aberrant kinetochore-microtubule attachments [106]. 

Recent studies have revealed that the human Aurora B kinase is preferentially modified by SUMO-2/3 at lysine 202 (K202) within its kinase domain (Table **1** and Fig. **2**) [84,85]. As the major SUMOylation site of human Aurora B, the K202 is located within a highly conserved sequence region (IHRDIKPEN), which is identical among the Aurora B proteins of different species ranging from yeast to human and also contains the SUMOylation consensus motif (IKPE) [84,85]. Although each of the five human PIAS family members (PIAS1, PIAS3, PIASxα, PIASxβ and PIASy) can interact with Aurora B, only PIAS3 efficiently stimulates the SUMO-2/3-specific modification of Aurora B *in vivo* [84]. Consistent with the PIAS3-specific stimulation of Aurora B SUMOylation, the GFP-tagged PIAS3 proteins have been found to be associated with kinetochores as paired foci on both sides of Aurora B during prophase and prometaphase [84]. The fluorescence signals of GFP-PIAS3 disappear on chromosomes during metaphase. Interestingly, a small portion of GFP-PIAS3 is also co-localized with Aurora B at the spindle midzone during anaphase [84]. Among the five FLAG-tagged mammalian SENPs (SENP1, SENP2, SENP3, SENP5 and mouse SENP2 isotype Smt3IP2/Axam2), only overexpression of FLAG-SENP2 in HeLa cells leads to a significant deSUMOylation of Aurora B [84]. Consistent with the previous finding [76], overexpression of FLAG-SENP2 does not affect Aurora B localization to inner centromeres during mitosis, indicating that SUMO-2/3 modification of Aurora B is not required for its localization to inner centromeres [84]. 

Depletion of Aurora B kinase by RNA interference results in the defect in chromosome alignment to metaphase plate and also the failure in cytokinesis, leading to accumulation of multinucleated cells [104]. Although the SUMOylation-null mutant of Aurora B (Aurora B^K202R^) has the same kinase activity as the wild-type Aurora B *in vitro*, the Aurora B^K202R^ mutant is unable to rescue the mitotic defects caused by RNAi-depletion of endogenous Aurora B, suggesting that SUMOylation of Aurora B is required for chromosome congression and cytokinesis [85]. Furthermore, the stable expression of the Aurora B^K202R^ mutant in cells with depletion of endogenous Aurora B also causes the failure of the CPC to be accumulated at inner centromeres during prometaphase and metaphase, the increased level of the CPC on chromosome arms, and the defect in centromeric function, which is indicated by the reduced phosphorylation of the Aurora B substrate CENP-A. This result suggests that SUMO modification of Aurora B might be required for the removal of the CPC from chromosome arms during prometaphase [85]. Moreover, it has been shown recently that SUMO-2/3 modification of Aurora B can greatly enhance its autophosphorylation* in vivo*, which is essential for its activation during mitosis [84]. This result supports a model that SUMOylation of Aurora B is a novel mechanism to regulate its kinase activity during mitosis [84].

Analysis of SUMOylation of the CPC in mammalian cells has revealed that its non-enzymatic subunit, Borealin, is preferentially modified by SUMO-2/3, and that the level of its SUMOylation in metaphase is higher than that in anaphase (Table **1** and Fig. **2**) [86]. Interestingly, Nup358/RanBP2 has been identified as the SUMO E3 ligase for Borealin both* in vitro *and *in vivo* [86]. Furthermore, the SUMO isopeptidase SENP3 has a specific interaction with Borealin *in vivo* and is responsible for deSUMOylation of Borealin [86]. However, SUMOylation of Borealin does not affect the assembly of the CPC as well as its localization at centromeres and spindle midzone [86]. Interestingly, the yeast Survivin homolog Bir1 has also been identified as a SUMO target, but the role of its SUMOylation is currently unknown [107]. 

### The CENP-H/I/K Complex at Inner Kinetochore

Studies of the mammalian SUMO isopeptidase SENP6 have shown that the inner kinetochore proteins CENP-H and CENP-I are specifically modified by polymeric SUMO-2/3 chains (Table **1** and Fig. **2**) [88]. The CENP-H/I/K complex consists of CENP-H, CENP-I and CENP-K and belongs to the constitutive centromere-associated network (CCAN), the so-called inner kinetochore. The CCAN is assembled onto and also associated with the CENP-A-containing chromatins throughout the cell cycle [108]. The CCAN consists of 16 proteins, which are classified into six different groups/ complexes including CENP-C, CENP-H/I/K, CENP-L/M/N, CENP-O/P/Q/R/U(50), CENP-T/W, and CENP-S/X [108]. Similar to the yeast Ulp2, SENP6 preferentially catalyzes deSUMOylation of polymeric SUMO-2/3 chain-modified substrates [27]. RNAi-depletion of SENP6 results in an accumulation of the polymeric SUMO-2/3 chain-modified CENP-H and CENP-I (CENP-H/I) during S phase, rather than in mitosis [9,88]. Because the CENP-H/I/K complex is recruited to the constitutive inner kinetochore structures during S phase, it has been hypothesized that SUMOylation of CENP-H/I promotes the assembly of inner kinetochores [9,88]. In SENP6-depleted cells, the poly-SUMO-2/3 chains on CENP-H/I are recognized by RNF4, the SUMO targeted ubiquitin ligase (STUbL), leading to polyubiquitination and proteasome-mediated degradation of CENP-H/I [88]. Furthermore, SENP6 depletion leads to the chromosome congression defect, the prolonged mitotic arrest, and the chromosome missegregation in mammalian cells [88]. 

In SENP6-depleted cells, both the CENP-H/I/K complex and the CENP-O complex (CENP-O/P/Q/R/U) are undetectable at the inner kinetochore plate [88]. The inner kinetochore localization of the CENP-O complex has been known to be completely dependent upon the CENP-H/I/K complex [109]. Therefore, the mislocalization of the CENP-O complex from the inner kinetochores in SENP6-depleted cells is mainly caused by the degradation of the CENP-H/I/K complex [88]. The depletion of CENP-H/I/K components can also lead to a decrease of the Ndc80/Hec1 and the Mis12 complexes at outer kinetochore [110]. The Ndc80/Hec1 complex and the Mis12 complex belong to the conserved KNL-1/Mis12 complex/Ndc80 complex (KMN) network and coordinate in promoting the outer kinetochore assembly and the kinetochore-microtubule attachment [111]. The mitotic defects in SENP6-depleted cells have been found to be very similar to those in the CENP-H/I/K-depleted cells, suggesting that SENP6 is a key regulator of inner kinetochore assembly by preventing the polymeric SUMO-2/3 chain modification of the CENP-H/I/K complex and thereby protecting CENP-H/I/K from RNF4-mediated degradation during S phase [9,88]. 

Because CENP-H/I are required for CENP-C localization to kinetochores, it would be very interesting to test whether SUMOylation of CENP-H/I plays a role in recruiting CENP-C as well as other proteins to kinetochores [112]. It has been well established that SUMO modification functions in enhancing the interactions between SUMO-modified proteins and other proteins containing SUMO-interaction motifs (SIMs), leading to the assembly of large protein complexes, such as PML nuclear body (PML-NB) [19,113,114]. One of the intriguing questions in the SUMO field is how overexpression of SUMO proteins suppresses the temperature sensitive phenotypes of the CENP-C mutant in both budding yeast and chicken cells [11,83]. The *S. cerevisiae* SUMO gene named *SMT3* was first identified in a genetic screen of high copy suppressors of the temperature sensitive mutations in *MIF2* gene, which encodes the yeast homolog of mammalian CENP-C [11]. Consistent with the finding in *S. cerevisiae*, the human SUMO-1 gene also suppresses the temperature-sensitive phenotype of the chicken CENP-C mutant in the chicken DT40 cells [83]. At the restrictive temperature, the chicken CENP-C mutant cells display multiple defects, including metaphase delay, chromosome missegregation, and cell cycle arrest at G1 phase [83]. Interestingly, CENP-C has been identified as a SUMO target *in vitro* [115]. Therefore, we hypothesized that SUMO-1 overexpression increases SUMO-1 modification of CENP-H/I and/or CENP-C mutant, which facilitates the interaction between CENP-H/I and CENP-C mutant, leading to a more effective assembly of CENP-C mutant onto kinetochores in the CENP-C mutant cells. The other possibility is that SUMOylation of CENP-C mutant directly facilitates the CENP-C mutant to re-gain its correct folding under the non-permissive temperature so that the SUMOylated CENP-C mutant is more efficiently targeted to kinetochores than the unmodified mutant. Consistent with this hypothesis, SUMO has been widely fused to many proteins to enhance the solubility of these proteins, leading to more efficient protein expression and purification in various protein expression systems [116-120].

### Nuf2, BubR1 and CENP-E at Outer Kinetochore and Fibrous Corona

**Fig. (3):**
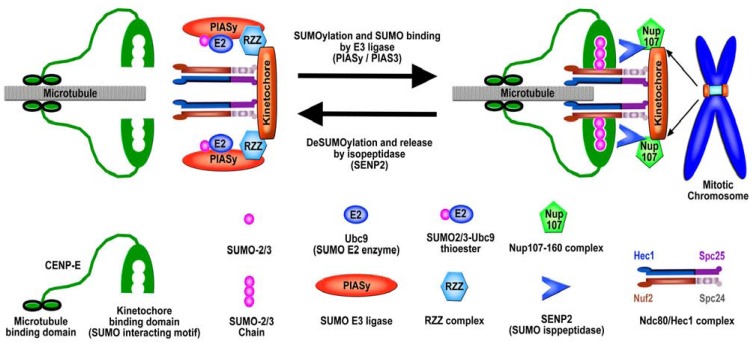
A model shows that polymeric SUMO-2/3 chain modification of kinetochore proteins regulates CENP-E localization to the kinetochores. The kinetochore-associated microtubule motor protein CENP-E contains the NH_2_-terminal microtubule binding domain, the rod domain, and the COOH-terminal kinetochore binding domain. CENP-E forms a dimer through its rod domain and is targeted to the kinetochore through its kinetochore binding domain. The kinetochore binding domain of CENP-E also contains a polymeric SUMO-2/3 chain interacting motif (notches) that is essential for CENP-E localization to the kinetochores. Furthermore, the known CENP-E-interacting proteins, Nuf2 and BubR1, have been shown to be specifically modified by SUMO-2/3 (purple circles). The Nuf2 is one of the subunits of the Ndc80/Hec1 complex, including Hec1, Nuf2, Spec24 and Spec25. Moreover, both SUMO E3 ligases, PIASy and PIAS3, are localized to unattached kinetochores during early mitosis. PIASy is targeted to the unattached kinetochores by the Rod/Zw10/Zwilch (RZZ) complex, recruits the SUMO thioester-loaded Ubc9 (SUMO E2 conjugating enzyme), and stimulates the poly-SUMO-2/3 chain modification of kinetochore proteins, such as Nuf2 and BubR1 (not shown here), leading to the kinetochore localization of CENP-E and the microtubule attachment to kinetochores. On the other hand, the SUMO isopeptidase SENP2 is hypothesized to be recruited to kinetochores through its interaction with the Nup107-160 complex, leading to the deSUMOylation of Nuf2 and BubR1 and the dissociation of CENP-E from kinetochores. Therefore, SUMOylation and deSUMOylation of the kinetochore proteins regulate the association and dissociation of CENP-E with kinetochores and thereby the microtubule attachment to the chromosome.

Studies of mitotic SUMOylation in mammalian cells have shown that the outer kinetochore protein Nuf2 and the fibrous corona-associated proteins BubR1 and CENP-E are specifically modified by SUMO-2/3 (Table **1** and Fig. **2**) [76]. Global inhibition of SUMOylation by overexpression of SENP2 causes a mitotic arrest at prometaphase due to the defect in targeting the microtubule motor protein, CENP-E, to kinetochores in mammalian cells (Fig. **3**) [76]. Furthermore, a SUMO-2/3 chain-interacting motif (SIM) has been identified at the C-terminal kinetochore-binding domain (tail domain) of CENP-E. This SIM is required for CENP-E binding to polymeric SUMO-2/3 chains *in vitro* and also essential for its targeting to kinetochores (Fig. **3**) [76]. Based on these findings, it has been hypothesized that the polymeric SUMO-2/3 chain modification of kinetochore proteins is required for targeting the polymeric SUMO-2/3 chain-interacting protein CENP-E to kinetochores (Fig. **3**) [76]. Consistent with this hypothesis, the two known CENP-E-interacting proteins, Nuf2 [121] and BubR1 [122,123], have been identified to be specifically modified by SUMO-2/3 *in vivo* (Fig. **3**) [76]. 

Recent studies have shown that SUMOylation of BubR1 is essential for its function during mitosis (Table **1** and Fig. **2**) [89]. As a key component of the spindle assembly checkpoint (SAC), BubR1 is localized to unattached kinetochores during early prophase and then disassociates from microtubule-attached kinetochores following chromosome congression to the metaphase plate [5]. BubR1 is predominantly modified by SUMO at Lysine 250 (K250), and its SUMOylation is strongly stimulated after a prolonged mitotic arrest caused by the treatment of either nocodazole or taxol [89]. Interestingly, SUMOylation of BubR1 does not regulate its activation and kinetochore localization [89]. However, ectopic expression of the SUMOylation-deficient BubR1 mutant causes a defect in the timely removal of the BubR1mutant from the kinetochore during metaphase for SAC inactivation, which leads to a delay of progression through mitosis, and also an increase of lagging chromosomes during anaphase [89]. These results indicate that BubR1 SUMOylation plays a critical role in its disassociation from kinetochores, the checkpoint inactivation for timely entry of anaphase, and accurate chromosome segregation. 

The Ndc80/Hec1 complex, including Ndc80/Hec1, Nuf2, Spc24 and Spc25 subunits, is one of the key components of the KMN network at the outer kinetochore plate and plays a major role in kinetochore-microtubule attachments [124,125]. It has been reported that Nuf2 can directly interact with CENP-E *in vitro *and is also required for targeting CENP-E to kinetochores in mammalian cells [121]. Although Ndc80 has been identified as a SUMO substrate in budding yeast, the functional significance of its SUMOylation is still unknown [107]. It would be very interesting to know whether the mammalian Ndc80 homolog, Hec1, is also a SUMO target *in vivo*. As a SUMO-2/3 substrate [76], the BubR1 kinase is a key component of SAC [96] and a CENP-E interacting protein at the fibrous corona [122,123]. Both BubR1 and CENP-E exhibit stronger localization at the kinetochores of unaligned chromosomes than those of aligned chromosomes [122,123]. In addition, BubR1 and CENP-E can be co-immunoprecipitated from HeLa cells [122,123]. Interestingly, CENP-E is also specifically modified by SUMO-2/3 at its tail domain *in vivo* (Fig. **2**), suggesting that SUMO-2/3 modification of CENP-E might also be important for its localization to kinetochores [76]. Because the SUMOylation sites at CENP-E tail domain have not been identified, the effect of CENP-E SUMOylation on its kinetochore localization cannot be directly tested by expression of the SUMOylation-deficient CENP-E mutant [76]. 

Overexpression of SENP2 results in a global loss of SUMO-1 and SUMO-2/3 modified proteins, except SUMO-1-modified RanGAP1 [76], which forms a stable complex with RanBP2/Nup358 and Ubc9 and is thereby protected from isopeptidase-mediated deSUMOylation [126]. Consistent with the global loss of SUMOylation, SENP2 overexpression also leads to the disappearance of the immunofluorescence staining of SUMO-2/3 on mitotic centromeres and kinetochores without affecting the SUMO1-RanGAP1 staining on mitotic spindles [76]. To further test whether the defect in CENP-E localization to kinetochores is a direct result of deSUMOylation other than a side effect caused by SENP2 overexpression, an independent approach was used to inhibit SUMOylation by RNAi-depletion of the only SUMO E2 enzyme Ubc9 [76]. Consistent with the defect caused by SENP2-mediated deSUMOylation, depletion of Ubc9 also results in a loss of global SUMOylation and a defect in targeting CENP-E to kinetochores, supporting the model that SUMOylation is essential for targeting CENP-E to kinetochores [76]. 

The chromosome congression defect in SENP2 overexpressing cells is almost the same as the defect caused by depletion or inhibition of CENP-E [122,127-129]. The chromosome congression defect is a failure in complete chromosome alignment to the metaphase plate with some chromosomes detected at the spindle pores. As a member of the kinesin-7 family and a plus end-directed motor protein, CENP-E is mainly associated with the kinetochore fibrous corona [130,131]. CENP-E functions in the kinetochore-microtubule attachment and plays a major role in chromosome congression from the spindle poles to the metaphase plate [128,132]. It has been shown that the loss of CENP-E at kinetochores in the SENP2 overexpressing cells is caused by the defect in targeting CENP-E to kinetochores other than by the degradation of CENP-E [76]. Unlike the RNAi-depletion of SENP6, SENP2 overexpression does not affect in the overall structure and function of kinetochores, because the centromere and kinetochore proteins (including Aurora B, Survivin, CENP-B, CENP-C, Hec1, CENP-F, Nup96 and Nup107) exhibit correct localization on mitotic chromosomes in SENP2 overexpressing cells [76,88]. In SENP2 overexpressing cells, the signals of spindle assembly checkpoint (SAC) proteins, including Bub1, BubR1 and Mad2, are largely undetectable on aligned chromosomes at the metaphase plate but accumulated on unaligned chromosomes at the spindle poles, indicating that the mitotic arrest caused by SENP2 overexpression is due to the activation of SAC. 

It is very interesting to reveal that the polymeric SUMO-2/3 chain modification can act as a specific signal distinct from the polymeric SUMO-1 chain and the monomeric SUMO modifications, in this case, for targeting the polymeric SUMO-2/3 chain-binding protein, CENP-E, to kinetochores [76]. In addition to CENP-E, the ubiquitin E3 ligase RNF4 has also been identified as one of the first polymeric SUMO chain-binding proteins and functions in targeting polymeric SUMO chain-modified substrates, including PML, CENP-H and CENP-I, for ubiquitination and ubiquitin-dependent proteolysis [88,133,134]. Interestingly, the RNF4 fragment containing four SUMO-interacting motifs (SIMs) has been successfully used for affinity purification and identification of more than 300 endogenous poly-SUMO-chain-modified proteins from cultured human cells [135]. Therefore, this novel strategy could be very useful to identify the endogenous mitotic targets specifically modified by poly-SUMO-chains in eukaryotic cells. Furthermore, the Zip1 protein, a component of the synaptonemal complex in *S. cerevisiae*, has also been demonstrated as one of the first SIM-containing proteins that exhibits a stronger binding to the polymeric SUMO chains than to the free SUMOs [47]. 

### RanGAP1 at Kinetochore Fibrous Corona

In vertebrates, SUMO-1 modification of RanGAP1 is required for the assembly of a highly stable multiprotein complex, called the RRSU complex, which consists of RanBP2/Nup358, RanGAP1-SUMO1 (SUMO-1-modified RanGAP1) and Ubc9 [8,26,69,90,136,137]. In interphase cells, the unmodified RanGAP1 is localized to the cytoplasm, whereas SUMO-1 modification of RanGAP1 facilitates RanGAP1 interaction with RanBP2/Nup358 and Ubc9 at the cytoplasmic filaments of the NPC, leading to the RRSU complex assembly [26,69,90,136,137]. During mitosis, the RRSU complex is localized to kinetochore fibrous corona (Table **1** and Fig. **2**) and mitotic spindles [8,25,81]. The association of the RRSU complex with kinetochores appears immediately after nuclear envelope breakdown and persists until the late anaphase [81]. RanBP2 is also required for targeting RanGAP1 to the NPC during interphase and to the kinetochore during mitosis [81,90-92]. 

The Ran GTPase controls many essential cellular processes, including nucleocytoplasmic transport, mitotic spindle assembly, and nuclear envelope formation [138]. Ran functions as a molecular switch by cycling between its GTP-bound and GDP-bound states. The switch between RanGTP and RanGDP is controlled by two regulatory proteins, RanGAP1 and RCC1 [139]. RanGAP1 functions in stimulating RanGTP hydrolysis to RanGDP in the cytoplasm during interphase, whereas the Ran guanine nucleotide exchange factor RCC1 is associated with chromatin throughout the cell cycle for RanGTP generation [139]. As a Ran binding protein, RanBP2 facilitates RanGAP1 in stimulating RanGTP hydrolysis to RanGDP [139]. Consequently, a steep gradient of RanGTP, which is much higher in the nucleus than in the cytoplasm, is established across the nuclear envelope during interphase. On the other hand, a diffusion-limited RanGTP gradient is formed with a higher concentration around the chromatins than at the cell periphery during mitosis in metazoan cells, in which the nuclear envelope disintegrates at late prophase. The concentration of RanGTP, which always increases around chromatins, has been hypothesized as a “genome-positioning system” called GPS in eukaryotic cells [138]. The GPS of RanGTP plays a central role in regulation of the functions of Ran. For instance, the nuclear export receptor called exportin CRM1 binds to its cargo in the presence of RanGTP in the nucleus. After exiting the nucleus, the RanGTP-CRM1-cargo complex is disassembled by RanGTP hydrolysis, leading to the release of cargo in the cytoplasm [139]. During mitosis, CRM1 functions in binding and delivering its mitotic cargoes to centromeres (Survivin), kinetochores (RRSU complex) and centrosomes (nucleophosmin) [138]. 

Interestingly, CRM1 delivers the RRSU complex as a cargo to the kinetochores through CRM1’s binding to the kinetochore-associated Nup107-160 complex during mitosis [140,141]. Interestingly, the kinetochore localization of the Nup107-160 complex is dependent on the Ndc80/Hec1 complex [140]. In addition, disruption of microtubule by nocodazole leads to the defect in targeting the RRSU complex to kinetochores [81]. Therefore, the kinetochore localization of the RRSU complex requires CRM1, RanGTP, the Nup107-160 complex and microtubule [81,141]. At the same time, disruption of the RRSU complex assembly at kinetochores results in defects in kinetochore-microtubule attachment, chromosome mis-alignment, and chromosome missegregation [92,138,141,142]. The RRSU complex has been demonstrated to be required for the kinetochore localization of CENP-E, CENP-F, Mad1, Mad2, Zw10 and Dynein [92]. 

## THE SUMO AND UBIQUITIN PATHWAYS MEET AT CENTROMERES AND KINETOCHORES

Several lines of evidence suggest that there is a crosstalk between the SUMO and ubiquitin pathways at centromeres and kinetochores. As discussed above, the human SUMO-1 gene was also identified as one of the seven genes that suppress the temperature-sensitive phenotype of the chicken CENP-C mutant by introducing a human cDNA library into the chicken CENP-C mutant DT40 cells [83]. Interestingly, this genetic screening has also identified a gene encoding the deubiquitinating enzyme Ubp16 (also called Ubp-M) that suppresses the temperature-sensitive phenotype of the chicken CENP-C mutant [83]. Recent studies have shown that Ubp16/Ubp-M is required for the cell cycle progression through mitosis [143]. Therefore, the above genetic findings strongly support a model that the SUMO and ubiquitin pathways coordinate in regulation of the functions of CENP-C at inner kinetochores.

The SUMO targeted ubiquitin ligases (STUbLs) are conserved from budding yeast to human and contain SIMs for recognition of the polymeric SUMO-chain-modified proteins [144]. The STUbLs specifically target polymeric SUMO-chain-modified proteins for ubiquitination and ubiquitin-dependent proteolysis, indicating that the polymeric SUMO-chains conjugated to their protein targets can serve as the upstream signals for polyubiquitination. Therefore, the STUbLs provide a direct link between the SUMO and ubiquitin pathways. Consistent with these findings, quantitative proteomic studies have also indicated that SUMO-2/3 modification of many protein substrates is tightly linked to the ubiquitin-proteasome system in mammalian cells [145,146]. The STUbL in budding yeast is a heterodimer called Slx5-Slx8, whereas the STUbL in fission yeast consist of two different heterodimers, Rfp1-Slx8 and Rfp2-Slx8 [144]. The mammalian STUbL called RNF4 (RING finger protein 4) contains four SIMs, whereas the yeast Slx5-Slx8, Rfp1-Slx8 and Rfp2-Slx8 contain one or two SIMs [133]. RNF4 binds strongly to polymeric SUMO-2 chain but weakly to SUMO-2 monomer and dimer *in vitro* [133]. Both SUMO-1 and SUMO-2/3 modified proteins are highly concentrated in the PML nuclear body (PML-NB) in interphase cells, whereas only SUMO-2/3 modified proteins are accumulated at inner centromeres and kinetochores from prophase to metaphase [76]. The accumulation of SUMO-2/3-modified proteins in PML-NB and at the centromere/kinetochore region suggests that these two subcellular domains might also contain the relative high concentrations of polymeric SUMO-2/3-chain-modified proteins as the potential targets for RNF4-mediated ubiquitination and ubiquitin-dependent proteolysis in mammalian cells. Consistent with this hypothesis, both PML and CENP-H/I proteins have been demonstrated to be poly-SUMO-chain-modified and then recognized by RNF4 for polyubiquitination and degradation [88,133]. Therefore, we proposed that the STUbLs mediate a direct crosstalk between the SUMO and ubiquitin pathways in control of the centromere/kinetochore assembly and disassembly.

As one of the subunits of the CPC, the yeast Survivin homolog Bir1 has been identified as a SUMO target [107]. At the same time, Survivin is modified by polyubiquitin chain through both Lys^48^ and Lys^63^ linkages in mammalian cells [147]. The Lys^48^ polyubiquitination functions in targeting protein substrates for ubiquitin-dependent proteolysis [148]. On the other hand, the Lys^63^ polyubiquitination of Survivin is required for targeting Survivin to centromere, whereas Lys^63^ de-ubiquitination of Survivin catalyzed by deubiquitinating enzyme hFAM is essential for the removal of Survivin from centromeres [147]. If SUMOylation of Survivin is also conserved in mammalian cells, it would be very interesting to test whether SUMOylation of Survivin functions in antagonizing its ubiquitination, which is similar to SUMOylation of IκBα [149] and PCNA [150,151], or in providing signals for RNF4-mediated ubiquitination and proteolysis. 

Both SUMOylation and ubiquitination regulate CENP-E localization to kinetochores [76,152]. As discussed above, SUMOylation is required for CENP-E localization to kinetochores [76]. At the same time, studies of ubiquitin-conjugating enzyme Cdc34/Ubc3 have shown that Cdc34/Ubc3-mediated ubiquitination inhibits CENP-E localization to kinetochores, leading to a mitotic arrest at prometaphase [152]. Interestingly, the mitotic arrest phenotype induced by overexpression of SENP2 is very similar to that caused by microinjection of recombinant Cdc34/Ubc3 proteins. In both cases, the mitotic arrest is not triggered by the ubiquitin-dependent proteolysis because inhibition of the proteasome-mediated degradation by MG132 does not rescue cells from the prometaphase arrest [76,152]. Therefore, the ubiquitination mediated by Cdc34/Ubc3 is likely to be the Lys^63^ polyubiquitination or monoubiquitination other than the Lys^48^ polyubiquitination. It would be very interesting to identify the mitotic target(s) of Cdc34/Ubc3 and also to investigate the effects of Cdc34/Ubc3-dependent ubiquitination on its substrate(s) in regulation of CENP-E localization to kinetochores. These studies will help us establish an important link between the SUMO and ubiquitin pathways in regulation of CENP-E localization to kinetochores during mitosis.

## SUMOYLATION IN CONTROL OF ACCURATE CHROMOSOME SEGREGATION

To ensure accurate chromosome segregation, the composition of kinetochores needs to be dynamically regulated during the cell cycle [5]. As discussed above, the constitutive centromere-associated network (CCAN) is associated with the CENP-A-containing chromatins at the inner kinetochore throughout the cell cycle [108]. However, the other kinetochore proteins are only temporally associated with kinetochores in a cell cycle-dependent manner. For examples, the Mis12 complex and KNL1 are assembled onto kinetochores during late G2 phase, whereas the Ndc80/Hec1 complex, the CPC complex, MCAK, Polo-like kinase, Bub1, BubR1, Bub3 and CENP-F are targeted to kinetochores during early prophase. On the other hand, CENP-E, Mad1, Mad2, dynein/dynactin, the RRSU complex and the Nup107-160 complex are localized to kinetochores during late prophase following the breakdown of nuclear envelope [5]. Furthermore, these transient kinetochore-associated proteins are shortly delocalized from kinetochores during late stages of mitosis. The CENP-E, Mad1, Mad2, BubR1 and dynein/dynactin are the first group of proteins disassembled from kinetochores during metaphase upon kinetochore-microtubule attachments, while the CPC complex, Polo-like kinase, Bub1, Bub3 and CENP-F disappear from kinetochores during the metaphase-anaphase transition. In addition, the Ndc80/Hec1 complex, RRSU complex and the Nup107-160 complex are delocalized from kinetochores during late anaphase, whereas MCAK, KNL1, and the Mis12 complex are disassociated from kinetochores during telophase [5]. Currently, little is known about the underlying mechanisms by which the temporal assembly and disassembly of kinetochore proteins are regulated, although posttranslational modifications including ubiquitination, phosphorylation and SUMOylation have been proposed to regulate this fundamental process. Simultaneously, we have little mechanistic understanding of how this transient kinetochore assembly is coordinated with other critical mitotic activities, such as kinetochore-microtubule attachments, SAC, and resolution of sister-chromatid cohesion. Recent advancements in our understanding of the SUMO pathway in mitosis have promoted us to propose a model that SUMOylation acts as a master regulator in control of multiple centromere/kinetochore activities including kinetochore assembly and disassembly, kinetochore-microtubule attachment, activation and inactivation of SAC, and decatenation of centromeric DNA.

Here, we would like to first use CENP-E as an example to elucidate the model that both SUMOylation and deSUMOylation regulate the assembly and disassembly of kinetochore proteins in vertebrates [76] (Fig. **3**). As a transient kinetochore-associated protein, CENP-E localizes to the fibrous corona immediately upon nuclear envelope breakdown during the late prophase, remains bound to the fibrous corona until chromosome congression to the metaphase plate, and then relocalizes to the spindle midzone following the onset of anaphase [153,154]. However, little is known about how the transient association of CENP-E with kinetochores is regulated. It has been hypothesized that SUMO E3 ligase(s) and isopeptidase(s) regulate the level of SUMO-2/3 modification on kinetochore-associated proteins and thus control the transient association of CENP-E to kinetochores through CENP-E’s SUMO-2/3-binding motif [76] (Fig. **3**). As discussed above, the two known-CENP-E interacting proteins BubR1 and Nuf2 have been demonstrated to be specifically modified by SUMO-2/3 *in vivo*. In addition, SENP2 overexpression does not affect the kinetochore localization of BubR1, Nuf2 and the other known CENP-E interacting protein CENP-F [76]. Furthermore, BubR1, Nuf2 and CENP-F are known to be required for targeting CENP-E to kinetochores [121,155], and they are also assembled onto kinetochores earlier than CENP-E [5,155]. Therefore, it would be interesting to test whether CENP-F and other known CENP-E interacting proteins, such as the kinetochore-associated microtubule motor protein KIF18A required for CENP-E localization to kinetochores [156], are also modified by SUMO-2/3 *in vivo*. It is highly possible that SUMO-2/3 modifications of multiple CENP-E interacting proteins simultaneously contribute to the recruitment of CENP-E to kinetochores. 

Although SUMO-2/3 modification has been known to play an essential role in regulation of CENP-E localization to kinetochores [76], little is known about how SUMO-2/3 modification of the kinetochore proteins, such as Nuf2 and BubR1, is regulated by SUMO E3 ligase(s) and isopeptidase(s) during mitosis. Although the SUMO isopeptidases SENP1 and SENP2 are both localized to the nucleoplasmic side of the NPCs during interphase [27], only SENP2 overexpression causes a mitotic arrest in mammalian cells [76]. It would be very interesting to test whether SENP2 is temporally targeted to kinetochores during mitosis to remove SUMO-2/3 from kinetochore proteins, such as Nuf2 and BubR1, leading to the delocalization of CENP-E from kinetochores. Consistent with this idea, a recent study has shown that SENP2 interacts with the Nup107-160 complex at the NPC during interphase [70]. Due to its association with kinetochore from late prophase to late anaphase [5,157], the Nup107-160 complex might facilitate SENP2 localization to kinetochores during mitosis (Fig. **3**). 

We would like to consider PIAS3 and/or PIASy as the E3 ligase(s) to stimulate SUMO-2/3 modification of kinetochore proteins for targeting CENP-E to kinetochores (Fig. **3**). Among the five vertebrate PIAS E3 ligases (PIAS1, PIAS3, PIASxα, PIASxβ and PIASy), PIAS3 is responsible for efficient SUMO-2/3 modification of Aurora B *in vivo* and is localized only to the unattached kinetochores during prophase and prometaphase [84]. On the other hand, PIASy plays a critical role in enhancing SUMO conjugation at centromeres/kinetochores and is the E3 ligase required for SUMO-2/3 modification of Topo IIα at mitotic centromeres in human cells and* Xenopus* egg extract [77,100,101]. Interestingly, PIASy is recruited to the centromere/kinetochore region by the Rod/Zw10/Zwilch (RZZ) complex, leading to the accumulation of SUMO-2/3 conjugates at the centromere region in *Xenopus* egg extract [103] (Fig. **3**). Therefore, it would be very interesting to test whether PIASy and/or PIAS3 are the E3 ligases for SUMO-2/3 modification of the kinetochores proteins, such as Nuf2 and BubR1, in regulation of CENP-E localization to kinetochores. 

Here we propose a model that the kinetochore-associated SUMO E3 ligases, such as PIASy and PIAS3, play a critical role in SUMO-2/3 modification at centromeres/kinetochores and thereby regulate multiple mitotic activities including the kinetochore assembly and disassembly, the kinetochore-microtubule attachments, SAC, and the resolution of centromeric DNA catenation in vertebrates. In this model, the localization of PIAS3 and PIASy to unattached kinetochores stimulates SUMO-2/3 modification of the centromere proteins, including Aurora B and Topo IIα [84,103], as well as the outer kinetochore proteins, including Nuf2 and BubR1(Fig. **3**). As one of the key components of SAC, the RZZ complex is associated with the unattached kinetochores to recruit the SUMO E3 ligase PIASy and other SAC proteins, such as Mad1, Mad2, and dynein/dynactin [103,158-160]. The PIASy-mediated SUMO-2/3 modification of Topo IIα at K660 inhibits the decatenation activity of Topo IIα, leading to a temporal block of the resolution of centromeric DNA until the inactivation of SAC at the onset of anaphase [87]. At the same time, the PIAS3-mediated SUMO-2/3 modification of Aurora B kinase at K202 facilitates the removal of the CPC from the chromosome arms from prophase to metaphase and also leads to the concentration of the CPC at the centromeric region of condensed chromosomes [84,85]. The concentrated centromeric localization of the CPC is critical for Aurora B kinase to destabilize and to correct the aberrant kinetochore-microtubule attachments by phosphorylating some key kinetochore proteins including Ndc80/Hec1 [85]. Furthermore, the PIAS3/PIASy-mediated SUMO-2/3 modification of kinetochore proteins such as Nuf2 and BubR1 at the unattached kinetochores facilitates CENP-E localization to those kinetochores, leading to the chromosome congression to the metaphase plate for the establishment of bi-oriented kinetochore-microtubule attachments [76] (Fig. **3**). Moreover, the bi-oriented kinetochore-microtubule attachments at the metaphase plate result in the release of the RZZ complex from the kinetochores and the inactivation of SAC [5,7]. The release of the RZZ complex causes the delocalization of PIASy from the kinetochores, leading to a decrease of SUMO-2/3 modification of centromere and kinetochore proteins including Topo IIα, Aurora B, Nuf2 and BubR1, due to the deSUMOylation activities of SENPs (Fig. **3**). Because SENP2 is in association with the Nup107-160 complex at the NPC during interphase [70], SENP2 might be recruited to kinetochores by the Nup107-160 complex during mitosis [5,157] to remove SUMO-2/3 from other centromere and kinetochore proteins (Fig. **3**). The decrease of SUMO-2/3 modification of kinetochore proteins, such as Nuf2 and BubR1, will lead to the delocalization of CENP-E from the kinetochores to the spindle midzone following the chromosome segregation during anaphase. At the same time, the loss of SUMO-2/3 modification of Topo IIα will cause the re-activation its enzymatic activity, leading to the decatenation of centromeric DNA. The Topo IIα-mediated decatenation of centromeric DNA along with the resolution of chromosome cohesion at the centromeres leads to the chromosome separation and segregation. 

## Figures and Tables

**Table 1.** The Known SUMO Targets at Centromeres and Kinetochores in Vertebrates

Locations	SUMO Targets	Organisms (GI number)	SUMOylation Sites	SUMO-1 or SUMO-2/3	SUMOylation Time	In Protein Complexes	Ref.
**Centromere**	**Aurora B**	Human (83776600)	K202	SUMO-2/3	Early Mitosis	CPC	[84,85]
	**Borealin**	Human (8922438)	Unknown	SUMO-2/3	Early Mitosis	CPC	[86]
	**Topo IIα**	Xenopus (148222806)	K660	SUMO-2/3	Early Mitosis	None	[82,87]
**Inner Kinetochore**	**CENP-H**	Human (12597655)	Unknown	SUMO-2/3	S Phase	CENP-H/I/K	[88]
	**CENP-I**	Human (41352697)	Unknown	SUMO-2/3	S Phase	CENP-H/I/K	[88]
**Outer Kinetochore**	**Nuf2**	Human (117968420)	Unknown	SUMO-2/3	Unknown	Ndc80/Hec1	[76]
**Fibrous Corona**	**BubR1**	Human (59814247)	K250	SUMO-2/3	Late Mitosis	None	[76,89]
	**CENP-E**	Human (71061468)	Unknown	SUMO-2/3	Unknown	None	[76]
	**RanGAP1**	Mouse (341941806)	K526	SUMO-1	Constitutive	RRSU	[25,26,90-92]

Note: K stands for a lysine residue. Ref. represents the corresponding reference(s) for each SUMO target. The RRSU complex consists of RanBP2/Nup358, RanGAP1-SUMO1 and Ubc9. The chromosomal passenger complex (CPC) contains Aurora B kinase, INCENP, Survivin and Borealin.
